# The Italian language postpartum specific anxiety scale [PSAS-IT]: translation, psychometric evaluation, and validation

**DOI:** 10.3389/fpsyt.2023.1208613

**Published:** 2023-08-09

**Authors:** Chiara Ionio, Giulia Ciuffo, Marta Landoni, Martina Smorti, Anna Maria Della Vedova, Paul Christiansen, Victoria Fallon, Sergio A. Silverio, Alessandra Bramante

**Affiliations:** ^1^CRIdee, Dipartimento di Psicologia, Facoltà di Psicologia, Università Cattolica del Sacro Cuore, Milan, Italy; ^2^Dipartimento di Patologia Chirurgica, Medica, Molecolare e dell'Area Critica, Università di Pisa, Pisa, Italy; ^3^Dipartimento di Scienze Cliniche e Sperimentali, Facoltà di Medicina e Chirurgia, Università Degli Studi di Brescia, Brescia, Italy; ^4^Department of Psychology, Institute of Population Health, Faculty of Health and Life Sciences, University of Liverpool, Liverpool, United Kingdom; ^5^Department of Women & Children’s Health, School of Life Course & Population Sciences, Faculty of Life Sciences & Medicine, King’s College London, London, United Kingdom; ^6^Policentro Donna, Milan, Italy

**Keywords:** anxiety, postpartum, scale development, maternal mental health, psychometrics, Italian population

## Abstract

**Introduction:**

While often positive, the lifecourse transition to motherhood is susceptible to the risk for developing mood disorders. Postpartum anxiety has often been overshadowed by other perinatal-specific mental health disorders, such as postpartum depression, and therefore has not been at the forefront or center of as much empirical study. This has meant there is a lack of effective and reliable tools with which to measure it, despite growing evidence suggesting its detrimental impact on mothers, their babies, wider family and social contacts, and on healthcare systems. This current study aimed to translate and validate the Postpartum Specific Anxiety Scale [PSAS] into the Italian language, and to validate the tool for its use in detecting anxiety specific to motherhood.

**Methods:**

The study (*N* = 457) comprised 4 stages: English-Italian translation and back-translation to obtain the Italian version [PSAS-IT]; a preliminary pilot study to adapt the PSAS to the characteristics of the Italian population; measurement invariance; and internal reliability of subscales.

**Results:**

The PSAS-IT demonstrates similar psychometric properties as the original English-language PSAS, with acceptable acceptability, construct and convergent validity, and internal consistency. Confirmatory factor analysis for multiple groups (Italy and United Kingdom) showed that the factor structure of the PSAS was valid for both groups [*χ*^2^ (2436) = 4679.481, *p* < 0.001, TLI = 0.969, CFI =0.972, RMSEA = 0.045, SRMR =0.064].

**Discussion:**

The resulting findings offer a reliable measure of postpartum anxiety in Italian language up to six months after birth.

## 1. Introduction

Postpartum mental health is increasingly topping the list of health concerns among Westernized countries. In high-income countries, up to 20% of women experience postpartum mental health problems (1). Postpartum depression has been the subject of research for decades, whereas anxiety symptoms have been largely ignored (2). However, because of its high incidence rates and impact on maternal and infant outcomes, postpartum anxiety is an essential topic for perinatal scientists and practitioners (3–5).

Some postpartum anxieties are a typical reaction to the birth of a child. During the postpartum period, women experience adaptive anxieties around adjusting to parenthood and caring for a newborn. However, Wisner (6) suggests postpartum anxiety becomes problematic when it consumes a significant proportion of daily life and impacts a mother’s ability to care for her infant. Research highlights maladaptive levels of postpartum anxiety affect between 11–17% of women (7). For this reason, careful assessment and use of validated screening tools are crucial for detecting and preventing maladaptive outcomes (6). Generally, anxieties are maternal- or infant-focused, but variables such as domestic responsibilities and financial concerns also contribute to many elements of worry. In addition, there is a significant co-occurance between anxiety problems and other mood disruptions during this time (8–10).

The National Institute of Health and Care Excellence (11) urged attention in 2014, recognizing the enormous burden of postpartum anxiety. Decreased breastfeeding (12, 13), poorer maternal–infant bonding (14, 15), decreased maternal sensitivity (16), poor attachment (17), abnormal neurodevelopment (5), and infant emotional and behavioral difficulties (4) are just a few of the adverse maternal and neonatal outcomes associated with postpartum anxiety.

From a clinical perspective, this is a significant omission, as there is growing evidence demonstrating postpartum maternal anxiety can have an increased risk for suicide and neonatal morbidity, both of which are associated with high healthcare costs (18). In Italy, a multi-center study conducted by the Italian National Institute of Health in August 2020 showed how the prevalence of postpartum anxiety was more than double the overall pooled prevalence of 15% (1 to 24 weeks postpartum) and 14.8% (> 24 weeks) observed in meta-analytic studies (8, 19). Despite recent and increasing scientific data supporting the need for early detection (20) and rapid treatment of maternal anxiety, anxiety in perinatal women in Italy generally remains unrecognized and untreated.

Most research on postpartum anxiety (4, 5, 21), relies on general measures of anxiety that can be psychometrically problematic. The State–Trait Anxiety Inventory [STAI; (22)] and the Generalized Anxiety Disorder-7 scale have identified and quantified postpartum anxiety [GAD-7; (23)]. These scales were developed for general adult populations but extrapolated for use in the postpartum period. Items on the STAI such as “I feel rested” may falsely increase anxiety scores because sleep disturbances are a natural part of parenting (24). On the other hand, broad-based evaluations do not consider particular mother and newborn issues; thus, low scores may not indicate a lack of symptoms (25).

A series of self-report questionnaires have been developed to measure specific anxieties related to the prenatal period which cannot be captured on general scales such as the Pregnancy Anxiety Scale [PAS; (26)], the Pregnancy-Related Anxiety Questionnaire [PRAQ; (27)], the revised PRAQ [PRAQ-R; (28)], and the Pregnancy-Related Anxiety Scale [PRAS; (29)]. Constructs examined include fear of childbirth, fetal health and well-being, having a physically or mentally impaired child, mother-infant bonding, relationship changes, and appearance changes. Studies using these measures have revealed two significant findings: (a) they predict prenatal outcomes better than general measures of anxiety (29); and (b) they differ qualitatively and quantitatively from general anxiety and depression indices (28). As a result, experts now consider pregnancy-related anxiety a separate entity from anxiety at other life stages (28). According to recent research, most postpartum women do not meet the diagnostic criteria for an anxiety disorder, but still suffer from clinically significant “maternally focused worry” (25).

The only questionnaire, to date, available for only postpartum anxiety is the Postpartum Specific Anxiety Scale [PSAS; (3)]. The PSAS is a 51-item measure of postpartum-specific anxiety which captures four types of postpartum anxiety:

1. Maternal competence and attachment anxieties (15-items);
2. Infant safety and wellbeing anxieties (11-items);
3. Practical infant care anxieties (7-items);
4. Psychosocial adjustment to motherhood (18-items).

Excellent reliability has been found within the four factors (Cronbach’s α ranged from 0.80 to 0.91), as well as across the overall scale (Cronbach’s *α* = 0.95). The English-language PSAS has been subject to modification first as a 12-item research short-form for use in global crises [PSAS-RSF-C; (30)] developed during the pandemic; and also as a 16-item research short-form [PSAS-RSF; (31)]. The scale has also received broad global interest and approved translations have been published in Chinese [PSAS-CN; (32)], Persian [PSAS-IR; (33), PSAS-IR-RSF; (34), PSAS-IR-RSF-C; (35)], French [PSAS-FR; (36)]; and Spanish [PSAS-ES; (37)]; with other translations currently ongoing in Brazilian Portuguese, Dutch, Palestinian Arabic, amongst others. In each country, the PSAS demonstrated good acceptability, validity and test–retest reliability. Along with good internal consistency on the global scale and of each of the factors. However, neither the validity nor the reliability of this scale has been tested in Italy.

## 2. Methods

This study aimed to validate an Italian version of the postpartum specific anxiety scale [PSAS-IT] and investigate its psychometric properties in order to have a sensitive instrument for detecting postnatal anxiety, thus responding to the demonstrated need for early screening and intervention in this country.

### 2.1. Ethics

All procedures of the study were approved by a local (Tuscany) Ethics Committee ‘Comitato Etico Area Vasta Nord-Ovest’ (ref:-CEAVNO N12749/2018) and by the University of Liverpool Research Ethics Committee (ref:-IPH/3964). Women who chose to participate in the study gave their informed consent. All procedures used in human subjects research were in accordance with the ethical requirements of the institutional and/or national research committee and the 1964 Declaration of Helsinki and its subsequent revisions or comparable ethical standards.

### 2.2. Participants

**Figure 1:**
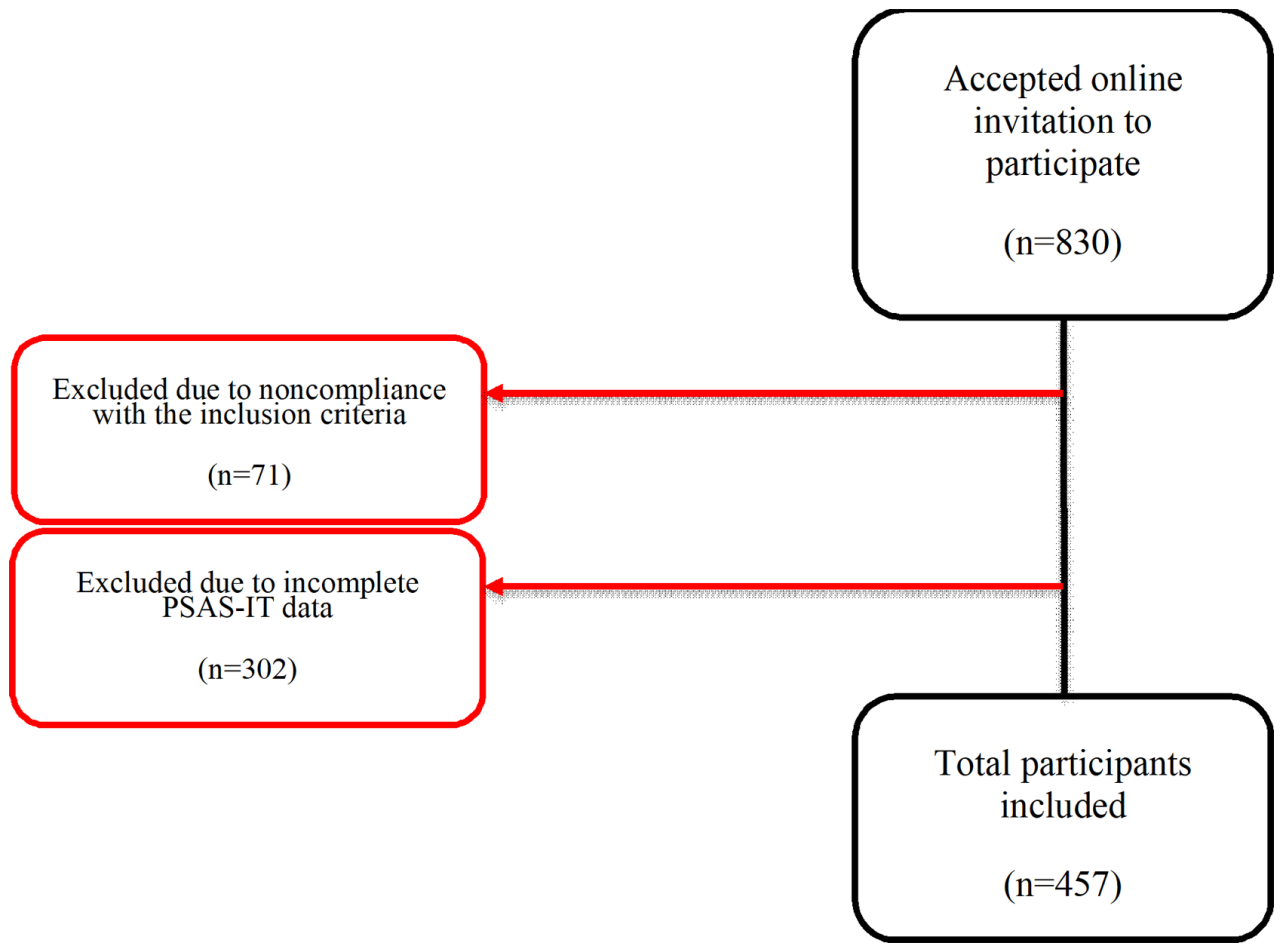
Phases of procedure.

Participants were mothers (*N* = 457) of infants aged between birth and six months postpartum. Of the 830 who took part, 373 were excluded from the final analyses as they had missing data on the PSAS. Women were selected for the study using non-probability (opportunity) sampling among those who had consented to participate in the study, understood Italian, and could communicate in Italian. The procedure for recruitment can be found in Figure 1.

The inclusion criteria were: women over the age of 18 years; good knowledge of the Italian language; no cognitive impairment; and no psychiatric disorders. Participants were excluded if they self-reported a history of mental illness and/or a traumatic event in the family history in the last six months. In addition, women with psychotic disorders or post-traumatic stress disorder [PTSD] were excluded because clinically diagnosed and/or managed mental illness and PTSD may alter postpartum-specific anxiety symptoms.

We calculated the minimum sample size to detect a clinically significant difference, assuming an effect size of 0.8 and a power of 0.95. The sample size for an effect size d is 0.8, an alpha error probability of 0.05 (2-tailed), and a power (1-probability of type II beta error) of 0.95 is 200 participants. Further, according to the literature, a sample size of 5 to 10 participants per item is necessary for factor analysis (38). With 51 items and 5 participants per item, a minimum of 255 women were required for an effectively powered study.

### 2.3. Translation process

After obtaining the necessary approvals from the PSAS Working Group, the original text was translated from English into Italian using accepted translation methods (30). This was done by three native Italian speakers with an excellent command of both languages and knowledge of the PSAS and perinatal mental health. The Italian versions were then back-translated from Italian into English by two experienced back-translators who were not involved in the previous step and who are experts in psychology and midwifery research respectively, but not familiar with the scale. They selected the most eloquent version of the Italian to translate. Finally, two further people familiar with the specific topic area evaluated the translated and back-translated versions to check for any errors. The most eloquent translations were chosen to back-translate, meaning the resultant PSAS-IT was made up of items from all three original translators.

### 2.4. Measures

In addition to the PSAS-IT, participants were asked for relevant personal details and presented with a battery of psychometric scales. Demographic information and psychometric scales used in this study emulated those which would be collected and used, respectively, in Italian healthcare service settings.

#### 2.4.1. Demographics

Demographic information was gathered about the participants, comprising: age, ethnicity, place of birth, country of residence, occupation, level of education, and marital status. The participants were also asked about their mental health, specifically about a clinical diagnosis of anxiety and depression. Infant-related demographic information included: age, weight, length, multiple birth status, birth order, gestational age, and feeding practices.

#### 2.4.2. The Edinburgh Postnatal Depression Scale

The EPDS (39, 40) is a self-report instrument consisting of 10 items to assess the extent of maternal depression in the postpartum period. Subjects are asked to indicate on a 4-point Likert scale how they have felt in the past week. The total score ranges from 0 to 30. The validated Italian version has demonstrated good validity and reliability (Cronbach’s α =0.7894), confirming the validity of EPDS in identifying postnatal depression. The best cut-off for clinically significant postnatal depression using the Italian-language EPDS has been reported between 9 and 10 (41).

#### 2.4.3. The Gneralized Anxiety Disorder – 7-item

The GAD-7 (23) is a 7-item instrument that provides rapid screening of generalized anxiety disorder. Participants are asked to indicate on a 4-point Likert scale whether they have suffered from anxiety in the past two weeks. The total score ranges from 0 to 21. The instrument has previously demonstrated good validity and reliability (Cronbach’s *α* = 0.89). According to Johnson (42), the optimal balance between sensitive and specificity for the GAD diagnosis was found at a cut-off point of ≥10.

#### 2.4.4. Postpartum Specific Anxiety Scale – Italian version

**Figure 2:**
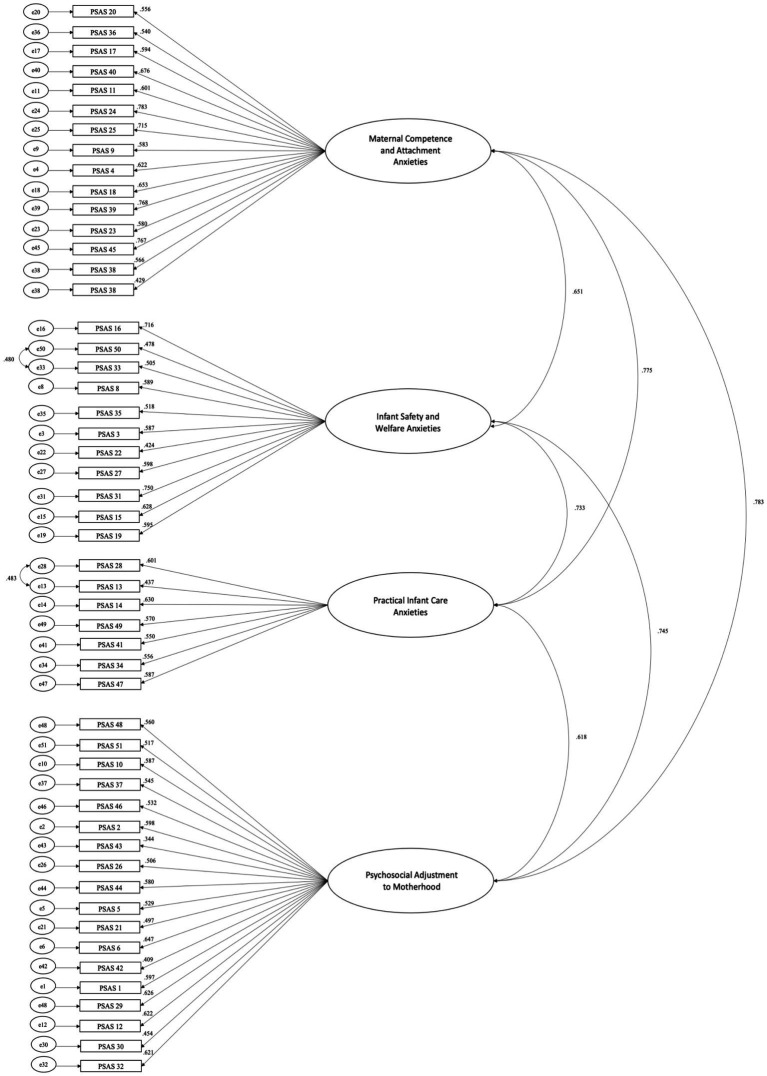
Standardized factor loadings.

The PSAS is a 51-item self-report instrument designed to examine the frequency of specific anxiety symptoms during the postpartum period. Women are asked to indicate on a 4-point Likert scale (ranging from 1 = never to 4 = almost always) how have they felt over the past week. The total score ranges from 51 to 204. A threshold score of 112 was suggested as detecting clinically significant levels of anxiety. Its structure comprises four factors: Maternal competence and attachment anxiety (15 items), Infant safety and welfare anxieties (11 items), Practical infant care anxieties (7 items), and Psychosocial adjustment to motherhood (18 items). Details about the items to sum for each subscale are presented in Figure 2. The scoring is reversed for the following questions: 2, 3, 5, 7, 9, 11, 12, 15, 18, 20, 24, 25, 29, 33, 34, 35, 37, 39, 40, 41, 42, 43, 44, 46, 48, and 51. The original version demonstrated excellent validity and reliability within the four factors (Cronbach’s *α* ranged from 0.80 to 0.91), as well as across the overall scale (Cronbach’s *α* = 0.95).

### 2.5. Procedure

Through on-line adverts with a link to Qualtrics software, mothers of infants between birth and six months postpartum were recruited for a study, between March and April 2022. Adverts were placed on parenting discussion forums in Italy, and social media sites. Each response was linked to a unique ID integrated into the survey program to maintain anonymity. The link to access the online survey remained active throughout the duration of the study until the follow-up phase.

An information sheet on the first page explained the objectives and methods of the study to participants. Mothers could withdraw from the study at any time without giving a reason, as participation was voluntary. Any distressing circumstance identified or request for support was forwarded to the study’s local Chief Investigator for discussion or referral to therapy. To this end, participants’ contact details were requested at the end of the survey. No link was made between participant data and contact information, and data were processed anonymously and identified only by a unique ID number. The database was stored on a secure server, and access to the information was limited to the research team members.

### 2.6. Statistical analyses

The statistical analysis of the study was performed with R version 4.0. A four-step procedure was used to assess the measurement invariance of the PSAS. For all analyses, data were fitted with a diagonally weighted least squares estimator because the PSAS is ordinal (1–4 points score), as recommended by Mîndrilã (43) and in accordance with the analysis strategy of Davies et al. (14). First, configuration invariance (i.e., whether the factor structure holds for both samples) was tested by fitting the PSAS factor structure with a country grouping variable. This model was evaluated using a series of fit indices following Hu and Bentler (44). The comparative fit index (CFI) and the Tucker-Lewis Index (TLI), with values of >0.95, were found to be good. For the root mean square error of approximation (RMSEA), values <0.06 were considered good, and for the standardized residual mean square error (SRMR), values <0.08 were considered a good fit (44). The configural model was then compared to the metric invariance model, which was the same model but with factor loadings fixed across groups (with intercepts allowed to vary). The validity of the metric model was assessed using the cut-off points described in Chen (45) given that we had equally sized groups and some mixed invariance. The criteria for metric invariance were CFI differences (∆CFI) < −0.01, ∆RMSEA<0.015 and ∆SRMR<0.03. The metric invariance model was then compared to the scalar invariance model, which assumes that the factor loadings and axis intercepts are assumed to be the same across our groups. The assessment was the same as for metric invariance, except that the SRMR cut-off was stricter with ∆SRMR<0.015 to indicate scalar invariance. Finally, we also tested strict invariance where both residuals and slopes and intercepts were assumed to be constant (comparing with the metric invariance model) using the same limit as in the previous model comparison.

Finally, we tested the internal reliability of each of the subscales in both the Italian and the British samples (separately). We used McDonald’s omega (ω), which, unlike the more commonly used Cronbach’s alpha, neither assumes tau equivalence nor represents a lower bound on estimates [see (46)]. In addition, we also calculate split-half reliability [Guttman λ4, see (47)]. In both cases, the values should be above 0.7.

## 3. Results

### 3.1. Sample characteristics

The age of the final sample of 457 mothers ranged from 18 to 33 (*M* = 21.46; SD = 3.02). Participants were predominately women from Italy (97.4%), married (55.1%), primiparous (69.4%), and in administrative professions (24.3%). Very few women had a current clinical diagnosis of anxiety (5.0%) or depression (1.8%) at the time of participation. The babies’ ages ranged from 1 to 27 weeks (*M* = 15.32; SD = 7.38). See Table 1 for full demographic details.

**Table 1:** Characteristics of the study sample (*N* = 457).

Maternal characteristic	Value	Infant characteristic	Value
Maternal age (mean years ± SD)	21.46 (3.02)	Infant age (mean weeks ± SD)	15.32 (7.38)
Country of Residence (N/%)		Infant birth weight (mean grams ± SD)	3032.60 (921.35)
Italy	445 (97.4)	Birth order (N/%)	
UK	1 (0.2)	1st	317 (69.4)
Germany	2 (0.4)	2nd	114 (24.9)
Spain	2 (0.4)	3rd	20 (4.4)
Other European	3 (0.6)	4th	5 (1.1)
Other Non-European	4 (0.8)	5th or more	1 (0.2)
Ethnicity (N/%)		Timing of birth (N/%)	
White	446 (97.6)	Premature (<37 weeks)	26 (5.7)
African	1 (0.2)	Early Term (>37 < 39 weeks)	105 (9.2)
Other	10 (2.2)	Full Term (39 weeks)	119 (26.0)
Marital Status (N/%)		Post Term (>40 weeks)	207 (45.3)
Married	252 (55.1)	Multiple birth (N/%)	
Co-habiting	202 (44.2)	Yes	5 (1.1)
Separated	1 (0.2)	No	452 (98.9)
Single	2 (0.4)	Current feeding method (N/%)	
Occupation (N/%)		Exclusively breastfeeding (100%)	288 (63.0)
Managers, Directors, Senior Officials	18 (3.9)	Predominantly breastmilk (over 80%) with a little formula milk (20%)	51 (11.2)
Professionals	87 (19.0)	Mainly breastmilk (50–80%) with some formula milk	19 (4.2)
Associate Professionals and Technical	25 (5.5)	A combination of both breastmilk (50%) and formula milk (50%)	26 (5.7)
Administrative and Secretarial	111 (24.3)	Mainly formula milk (50–80%) with some breastmilk	13 (2.8)
Skilled Trade	68 (14.9)	Predominantly formula milk (over 80%) with a little breastmilk (20%)	10 (2.2)
Caring, Leisure and Other Service	38 (8.3)	Exclusively formula feeding (100%)	50 (10.9)
Sales and Customer Service	40 (8.8)		
Process, Plant and Machine Operatives	20 (4.4)	Maternal anxiety	Mean (±SD)
Housewife	25 (5.5)	Overall PSAS	108.39 (21.97)
Not in Paid Occupation	3 (0.7)	PSAS Factor 1	30.89 (6.49)
Unemployed	22 (4.8)	PSAS Factor 2	21.29 (5.06)
Education Attainment (N/%)		PSAS Factor 3	15.81 (3.65)
Middle school diploma	22 (4.8)	PSAS Factor 4	40.38 (9.16)
Secondary school	158 (34.6)		
Undergraduate education	210 (46.0)	Maternal mental health	Mean (±SD)
Postgraduate education	48 (10.5)	Overall EPDS (*n* = 275)	9.12 (5.38)
Other qualification	19 (4.2)	McDonald’s Omega = 0.885	
Current Diagnosis of Anxiety (N/%)			
Yes	23 (5.0)	Overall GAD-7 (*n* = 270)	6.77 (4.60)
No	431 (94.3)	McDonald’s Omega = 0.880	
Prefer not to say	3 (0.7)		
Current Diagnosis of Depression (N/%)			
Yes	8 (1.8)		
No	448 (98.0)		
Prefer not to say	1 (0.2)		

### 3.2. Configural invariance

Confirmatory factor analysis for multiple groups (Italy and United Kingdom) showed that the factor structure of the PSAS was valid for both groups (*χ*^2^ (2436) = 4679.481, *p* < 0.001, TLI = 0.969, CFI =0.972, RMSEA = 0.045, SRMR =0.064). See Table 2 for factor loadings and Figure 2. See Table 3 for characteristics of the Italian study sample compared to the characteristics of the UK study sample.

**Table 2:** Minimum and maximum standardized item loads for Italian and UK data.

	Italy		UK	
	Min	Max	Min	Max
Factor 1	0.27	0.68	0.32	0.76
Factor 2	0.46	0.70	0.42	0.70
Factor 3	0.41	0.64	0.52	0.73
Factor 4	0.38	0.69	0.45	0.77

All *p* values < 0.001.

**Table 3:** Italian and UK study sample characteristics.

	Samples	Mean	SD	*t*	*p* value
Maternal Age	Italy	21.46	3.02	23.147	<0.001
	UK	14.95	5.51		
Baby Age	Italy	15.32	7.39	−15.074	<0.001
	UK	25.55	15.01		
PSAS Factor1	Italy	30.88	6.49	3.724	<0.001
	UK	29.17	8.90		
PSAS Factor2	Italy	21.28	5.06	−9.688	<0.001
	UK	24.70	6.69		
PSAS Factor3	Italy	15.81	3.66	4.002	<0.001
	UK	14.85	4.27		
PSAS Factor4	Italy	40.38	9.17	−5.034	<0.001
	UK	43.33	9.97		

### 3.3. Metric invariance

The configural invariance model was compared to the metric invariance model (assuming equal loadings in all groups). The difference between the two models did not exceed the thresholds for ∆RMSEA = 0.013 or ∆SRMR =0.009, although it was just above the thresholds for ∆CFI = −0.02. Because two of the fit indices showed metric invariance and one was just above the threshold, we did not consider cross group loading, although a discussion of the items that must vary across groups to produce a ∆CFI < −0.01 can be found in S1.

### 3.4. Scalar invariance

The metric invariance model was then compared to the scalar invariance model (assuming equal loadings and intercepts between groups). The difference between the two models did not exceed the cut offs for ∆RMSEA = 0.005, ∆SRMR =0.004, although it was just above the limit for ∆CFI = −0.011. Again, two of the fit indices found were scalar invariant and the CFI was just above the limit, so we did not allow for intercept variation when comparing with strict invariance. In particular, variation of the intercept of PSAS14 resulted in a ∆CFI < −0.01.

### 3.5. Strict invariance

The scalar invariance model was then compared to the strict invariance model (assuming equal loadings, intercepts and residuals in all groups). The difference between the models did not exceed the limits for ∆RMSEA = 0.001, ∆SRMR =0.002 or ∆CFI = −0.003. See Table 4 for McDonald’s Omega and Guttman’s lambda.

**Table 4:** McDonald’s Omega and Guttman’s lambda for Italian and UK samples.

Factor	Italian		UK	
	ω	λ4	ω	λ4
1	0.88	0.92	0.92	0.92
2	0.90	0.91	0.90	0.91
3	0.72	0.71	0.83	0.80
4	0.80	0.82	0.86	0.87

### 3.6. Convergent validity

To assess the convergent validity of the PSAS-IT, Pearson rank correlation analyses were performed with other recognized anxiety and depression screening instruments (EDPS and GAD-7). The correlation coefficients obtained showed a strong and favorable relationship between the total scores of PSAS-IT and the anxiety scores of the GAD-7 and with the EPDS scale scores, demonstrating high convergent validity – see Table 5.

**Table 5:** Pearson rank correlations between the PSAS and other validated measures of anxiety and depression.

	GAD-7	EPDS
PSAS	0.642*	0.721*

^*^p < 0.01 (one tailed).

## 4. Discussion

### 4.1. Summary of main findings

The original PSAS was adapted to the characteristics of the Italian population using a pilot study and psychometric evaluations which assessed the translated instrument’s acceptability, validity, and measurement invariance. As a result, the psychometric properties of the PSAS-IT were similar to those of the original English version, showing acceptable acceptability, construct and convergent validity, and internal consistency.

Our results demonstrate the data fit the expected factorial structure well and the model is entirely invariant between the two languages (evidence of generalizability). Configurable invariance means the theoretical construct has the same subscales and item-factor configurations in both groups. In addition, two of the fit indices used to test metric invariance showed no significant differences between the two groups, suggesting each item represents the construct similarly in Italy and the United Kingdom.

As with metric invariance, two of the fit indices used to test scalar invariance showed no significant differences between the two groups, indicating participants in the two groups tended to score the same on the items. In particular, the variation in the intercept of PSAS14 (*“Mi sono preoccupata di far avere al mio bambino la sua routine”*) yielded a ∆CFI < −0.01. Finally, strict invariance suggests that the two groups have the same variance in item errors.

The results are also evidence of good reliability. In particular, we checked whether the items within the same factor were coherent in the Italian and British samples (internal consistency). As expected, all four factors had values above 0.7 in both samples.

The PSAS-IT showed a positive relationship with previously validated and commonly used measures of anxiety and postpartum depression, including the GAD-7 and EPDS scales, indicating high convergent validity, in a similar fashion to Fallon et al. (13) and Silverio et al. (30), as well as the other PSAS validations [i.e. (32–37)]. Overall, we conclude the PSAS-IT results are valid and reliable.

As for internal consistency and reliability, previous validation studies have shown that both internal consistency and reliability are good. Indeed, the internal consistency of the four factors in the French version was good to excellent (Cronbach’s alpha coefficients ranged from 0.77 to 0.88), indicating a high reliability of the PSAS-FR. In the Iranian version, the reliability of the instrument was almost identical to the British version, showing that the types of anxiety experienced by women are the same across income levels.

Compared with other validated measures of general anxiety, our results demonstrate the utility of using PSAS-IT, an instrument that identifies clinical anxiety in new mothers that occurs only in the postnatal period.

### 4.2. Strengths, limitations, and future directions

This is the first study to investigate the psychometric capabilities of the PSAS in an Italian context. Including women who gave birth vaginally or by cesarean section and the random selection are two significant advantages of this study. In this study, data were collected online, as in Fallon et al. (3), which means less control of the sampling process. For future studies, it would be necessary to compare the performance of PSAS-IT with other samples of mothers, including those with low social support, high social complexity, and multiple disadvantages, or with a personal or family history of mental illness, as our sample is not sufficiently representative of at-risk populations. In addition, because women with a history of mental illness were not included in the study, the results are less generalizable to clinical populations. Finally, the sampling for this study took place during the COVID-19 pandemic, which could affect the results.

### 4.3. Conclusion

The PSAS constitutes to date the only reliable measure for postpartum-specific anxiety also in the Italian context. The tool is quick and easy to implement, which makes its use recommendable for early identification of postpartum anxiety. Given the adverse maternal and neonatal outcomes due to postpartum anxiety, clinicians and researchers should use PSAS in order to provide early intervention.

## Data availability statement

The raw data supporting the conclusions of this article will be made available by the authors, without undue reservation.

## Ethics statement

The studies involving human participants were reviewed and approved by University of Pisa (ref:- N12749/2018) and University of Liverpool (ref: IPH/3964) Research Ethics Committees. The participants provided their written informed consent to participate in this study.

## Author contributions

AB, CI, MS, AMDV, SAS, and VF: conceptualization and investigation. CI, ML, GC, and PC: methodology and formal analysis. PC: software. SAS, VF, and AB: validation. CI and MS: resources and project administration. ML and GC: data curation. CI, ML, and GC: writing – original draft. AMDV, MS, AB, PC, VF, and SAS: writing – review and editing. ML, PC, GC, and CI: visualization. CI, MS, AB, SAS, and VF: supervision. All authors contributed to the article and approved the submitted version.

## Conflict of interest

The authors declare that the research was conducted in the absence of any commercial or financial relationships that could be construed as a potential conflict of interest.

The reviewer MG declared a past co-authorship with the authors SAS, VF, PC, AB, AMDV to the handling editor.

## Publisher’s note

All claims expressed in this article are solely those of the authors and do not necessarily represent those of their affiliated organizations, or those of the publisher, the editors and the reviewers. Any product that may be evaluated in this article, or claim that may be made by its manufacturer, is not guaranteed or endorsed by the publisher.
